# A pragmatic examination of active and passive recruitment methods to improve the reach of community lifestyle programs: The Talking Health Trial

**DOI:** 10.1186/s12966-017-0462-6

**Published:** 2017-01-19

**Authors:** Paul Estabrooks, Wen You, Valisa Hedrick, Margaret Reinholt, Erin Dohm, Jamie Zoellner

**Affiliations:** 1 Department of Health Promotion, Social and Behavioral Health, University of Nebraska Medical Center, 984365 Nebraska Medical Center, Omaha, NE 68198-4365 USA; 2 Department of Applied and Agricultural Economics, Virginia Tech, 304 Hutcheson Hall, Virginia Tech, Blacksburg, VA 24061 USA; 3 Department of Human Nutrition, Foods, and Exercise, Virginia Tech, 335A Wallace Hall, Virginia Tech, Blacksburg, VA 24061 USA; 4 Department of Human Nutrition, Foods, and Exercise, Virginia Tech, 1981 Kraft Drive, 1031 ILSB, Blacksburg, VA 24060 USA

**Keywords:** Beverages, Behavioral research, Randomized controlled trial, Rural population, Reach, Representativeness

## Abstract

**Background:**

A primary challenge for behavior change strategies is ensuring that interventions can be effective while also attracting a broad and representative sample of the target population. The purpose of this case-study was to report on (1) the reach of a randomized controlled trial targeting reduced sugary beverages, (2) potential participant characteristic differences based on active versus passive recruitment strategies, and (3) recruitment strategy cost.

**Methods:**

Demographic and recruitment information was obtained for 8 counties and for individuals screened for participation. Personnel activities and time were tracked. Costs were calculated and compared by active versus passive recruitment.

**Results:**

Six-hundred and twenty, of 1,056 screened, individuals were eligible and 301enrolled (77% women; 90% white; mean income $21,981 ± 16,443). Eighty-two and 44% of those responding to passive and active methods, respectively, enrolled in the trial. However, active recruitment strategies yielded considerably more enrolled (active = 199; passive = 102) individuals. Passive recruitment strategies yielded a less representative sample in terms of gender (more women), education (higher), and income (higher; p’s <0.05). The average cost of an actively recruited and enrolled participant was $278 compared to $117 for a passively recruited and enrolled participant.

**Conclusions:**

Though passive recruitment is more cost efficient it may reduce the reach of sugary drink reduction strategies in lower educated and economic residents in rural communities.

**Trial registration:**

Clinicaltrials.gov; ID: NCT02193009, July 2014, retrospectively registered.

## Background

Sugar sweetened beverages (SSB) are ubiquitous and contribute to a large proportion of energy intake in the United States [1] and evidence suggests that the low satiety provided by SSB may further increase caloric intake from other sources [2]. In addition to adversely impacting energy balance, the regular consumption of sugary drinks contributes to childhood and adult obesity, the development of metabolic syndrome and type 2 diabetes, and emerging data suggests that high SSB consumption may contribute to hypertension, inflammation, and heart disease [2–4]. A recent review of literature found consistently that focusing on SSB reduction by encouraging alternative low or no-calorie beverages was successful in facilitating weight change and improving other biological markers of metabolic health [5]. More recently still the Talking Health randomized controlled trial demonstrated effectiveness of group-based and automated telephone counseling in decreasing participant SSB consumption when compared to a match contact, physical activity control condition [6].

A primary challenge for SSB behavior change strategies is ensuring that interventions can be effective while also attracting a broad and representative sample of the target population. Indeed, the public health impact of any behavioral intervention may be operationalized as reach by effectiveness [7, 8]. For community organizations that would ultimately implement effective SSB reduction strategies, there is a need to understand how best to engage participants and maximize reach [8]. From a pragmatic perspective simply understanding the total yield from a recruitment strategy is insufficient [9]. Other factors are also salient such as the cost of recruitment and the representativeness of participants that are engaged through different recruitment strategies to ensure key subgroups in the population are not under-represented (e.g., those from lower SES, less educated, and minority groups [9–11]). This is not an insignificant point for those interested in eliminating health disparities. Designing and testing interventions that systematically (though likely unintentionally) exclude participants from sub-populations that experience disparities could result in interventions that are effect for those that need them the least, and worse, are ineffective for those that need them the most—further exacerbated existing disparities.

Unfortunately, across SSB and other lifestyle intervention studies, few report on the methods used to recruit participants or on the representativeness of the sample when compared to a defined target population [12]. Lam and colleagues systematic review of recruitment strategies for young adult participation in lifestyle interventions for the prevention of weight gain is a good example. When completing the data abstraction on recruitment strategies they found that 23 of the 25 articles had insufficient information to fully describe the recruitment processes used in the studies [12]. The recommendations derived from the systematic review were for researchers to provide detailed information on recruitment strategies and report on the representativeness of participants based on the target population characteristics [12].

Across behavioral intervention literature researchers have provided information distinguishing between passive and active recruitment strategies [12–19]. Passive recruitment strategies include those without direct interaction with potential participants (e.g., flyers, newspaper ads, targeted mailings). Active recruitment strategies include those with direct interaction with potential participants (e.g., outreach telephone calls, presentations at locations where the target population resides or aggregates). Within this literature it appears that passive recruitment strategies yield a higher number of participants, but a lower proportion of those exposed to recruitment when compared to active recruitment. Conversely, active recruitment methods appear to recruit a higher proportion of those exposed to recruitment strategies, but a lower total number of participants. The degree to which passive versus active strategies produce more representative samples, and at what cost, is less clear in that only a small proportion of studies report on these factors [13]. In those that have examined representativeness, the findings suggest that active recruitment strategies engage a sample that more closely aligns with the characteristics of the target population [3, 9, 15, 17, 20, 21]. While no known studies have compared active versus passive recruitment costs, in general, recruitment costs range around $400-$500 per recruited and randomized participant [19, 21] when considering all material and personnel costs, highlighting the need to explore cost-efficient recruitment strategies.

The purpose of this case-study was to address the current gap in the literature related to the reporting of reach—conceptualized as the number and representativeness of participants [22] - of a randomized controlled trial targeting reduced SSB intake in a rural 8 county region [6]. A second purpose was to examine differences between participants that were identified and enrolled in the trial based on active versus passive recruitment strategies. We hypothesized that passive strategies would engage participants that were older more likely to be women with higher average incomes and education when compared to participants engaged through active strategies [6]. As community organizations differ in resources from county to county [23], we also hypothesized that each county would likely use both active and passive strategies, but would also have a unique final recruitment strategy [23]. As such, we examined the number and representativeness of participants recruited from active versus passive strategies across counties. A third purpose was to assess recruitment strategy cost to provide pragmatic information necessary for research and community decision-making related to future recruitment for community behavior change interventions/programs. We hypothesized that passive recruitment methods would be more cost-efficient than active strategies [16, 18, 24].

## Methods

The presented data were collected as a case-study of recruitment procedures, costs, and outcomes of the Talking Health randomized-controlled trial [6, 25]. Talking Health was a six-month, community based, trial that examined the effectiveness of a multicomponent SSB reduction intervention when compared to a matched-contact control group targeting physical activity behaviors. The SSB intervention included 3 small group sessions and telephone support (1 live call and 11 automated counseling calls) over a 6-month period. The intervention content was developed using the Theory of Planned Behavior and strategies to increase the likelihood that participants with limited health literacy would obtain, process, and implement the information and skills necessary to reduce SSB. The target population included adult (>18 years) residents in rural, southwest Virginia who had access to a telephone, consumed 200 or more SBB kcals/day, and reported no contraindications for physical activity (due to matched contact control condition).

All potential participants were screened for eligibility using items from the Beverage Intake Questionnaire (7 SSB items) [26], Stanford Leisure-Time Activity Categorical measure (1 item) [27], a health literacy screener (3 items; scale score ranging from 5 to 15 reflecting highest to lowest likelihood of limited health literacy, respectively [28–30]), and a demographic survey (11 questions). During the screening process, the location/method of recruitment was recorded (with the exception of 41 individuals from the first two cohorts who were screened before this aspect of the study’s protocol was established).

### Recruitment methods

Individuals were recruited and screened for eligibility in eight counties across rural Southwest Virginia (i.e., Lee, Giles, Pulaski, Washington, Grayson, Wise, Wythe, and Montgomery) from April 2012–May 2014, with an average 4 to 6-week window of recruitment for each county. The Index of Medical Underservice (IMU) ranged from 51-61, with all eight counties being federal designated as medically underserved areas defined as scores less than 62. Population density ranged from 48.0-242.5 residents per square mile (Table 1). The goal of the overall study recruitment strategy was to accrue the necessary number of participants per county while also explicitly focusing on strategies that would increase the likelihood of a representative sample—all while engaging community organizations or settings that could potentially be involved in taking an SSB intervention to scale. As such, each county reflected a distinct study cohort and recruitment methods were tailored to community organizations that were available to support recruitment activities. Specific strategies were tailored by discussing the best locations where participants may aggregate with the community partners and then locations were matched with time and availability of the community partner to be engaged. This resulted in a variety of active and passive recruitment strategies across the eight study cohorts (Table 1). Participants were documented as being recruited via passive or active strategies in all but one county (the first study cohort).

**Table 1:** Descriptive information on participating counties, recruitment strategies used, and number of residents screened, eligible, and enrolled

County number	Lee	Giles	Pulaski	Washington	Grayson	Wise	Wythe	Montgomery	Total
Screened, *n*	65	96	178	117	115	158	193	134	1,056
Eligible, *n* (%)	54 (83.1)	59 (61.4)	91 (51.1)	75 (64.1)	78 (67.8)	76 (48.1)	105 (54.4)	82 (61.2)	620 (58.7)
Enrolled, *n*, (% of eligible)	41 (75.9)	31 (52.5)	53 (58.2)	32 (42.7)	35 (44.9)	32 (42.1)	40 (38.1)	37 (45.1)	301 (48.5)
Active, *n* (%)	20 (48.8)	21 (67.7)	34 (64.2)	27 (84.4)	17 (48.6)	22 (68.8)	30 (75.0)	28 (75.7)	199 (66.1)
Passive, *n* (%)	21 (51.2)	10 (32.3)	19 (35.8)	5 (15.6)	18 (51.4)	10 (31.2)	10 (25.0)	9 (24.3)	102 (33.9)
Primary active RS:	extension agents	free clinic	extension agent, retail shops	free clinics, Health Department/WIC	extension agent, Health Department/ WIC	Health Department/WIC, retail stores	Health Department, free clinic, extension agent	Health Department, free clinic	
Secondary active RS	none	daycare, festivals and community events	festivals and community events, community college	Head Start, community events	Head Start, Retail store, Community events, Daycare	festivals, RAM health clinic, Head Start	Head Start, community events	extension agent	
Passive RS	newspaper ad, flyers	newspaper ad, flyers	newspaper ad, flyers, postcard mailings	flyers, postcard mailings	newspaper ad, flyers, postcard mailings, emails	newspaper ad, flyers, postcard mailings, emails	newspaper ad, postcard mailings, flyers	flyers, emails	
MUA	61.6	60.8	60.7	56.1	51.0	61.1	61.0	60.1	
PD (per sq ml)	58.5	48.0	105.8	97.0	34.8	102.3	62.9	242.5	

*RS* Recruitment Strategy, *MUA* Medically Underserved Area, *PD* Population Density (per square mile)

### Community organizations and personnel

Active recruitment strategies (described below) were implemented by university-affiliated research assistants and hired local community research assistants or community champions. The primary community organization involved across a number of counties was Virginia Cooperative Extension, a system initially developed to help translate agricultural research into local farming practice that expanded to include community nutrition outreach over 60 years ago [31]. Local cooperative extension professionals were the primary community-based recruitment personnel in four counties and secondary community-based recruitment personnel in another (Table 1). Public health professionals from local health departments including Women, Infants, and Children (WIC) clinics that provide services for lower SES groups in the counties also played a primary role in 5 counties. Other community champions that supported recruitment strategies were retail shop owners or employees, health professionals from free clinics, Head Start and other early childhood education professionals, pastors from local churches, and local community college faculty and staff.

### Active strategies

Active recruitment strategies were operationalized as those that included direct interaction with potential participants via person-to-person telephone outreach, presentations at locations where the target population resided or aggregated, or in-person face-to-face meetings. Table 1 gives an overview of the active recruitment locations of implementation across the counties. Recruitment efforts in each county had a primary and secondary active recruitment strategy. Primary strategies included visits to permanent county locations where a representative or higher proportion of under-represented (i.e., lower income and education levels) frequented. Examples include strip malls, clinics, health departments, and apartment complexes. Visits most often included setting up a study booth to describe the project and engage local residents. The booth was staffed by both research assistants from the study team and community personnel. Secondary active recruitment strategies included research assistant attendance at community festivals and events or spending a brief period of time in retail stores, early childhood education centers, or other permanent county locations.

### Passive strategies

Passive recruitment strategies were operationalized as those that did not include direct person-to-person interaction with potential participants. These strategies included community flyers (8 counties), newspapers ads (6 counties), targeted postcard mailings (5 counties), and email listserves (2 counties). A commercial firm generated the mailing list for one county, while the remainder were provided by community-based recruitment personnel. The primary messages on print materials included an invitation to participate in a research study to learn about health, the ease with which the study could be joined (e.g., “you are just one call away from a healthier you”), and the benefits of participating in the study (e.g., free health screenings, group classes and telephone support).

### Cost tracking

Recruitment costs were operationalized as the human resources, materials, and transportation used in each county to engage potential participants. Human resource tracking was primarily related to active recruitment strategies and included university-affiliated research assistant time, community-based recruitment personnel time, and travel time to recruitment events. Community-based recruitment personnel hours were tracked via submitted time cards for payment, and payment ranged from $18-$20/h. University-affiliated research assistants reported time spent on recruitment activities in real time using google-docs. Community-based recruitment personnel costs were calculated using $20/h and research assistant costs were calculated using $25/h. Material costs were primarily associated with passive strategies and included newspaper advertisements and the cost of mailings. In addition, we tracked human resource cost related to participant screening for eligibility. Finally, transportation costs as well as other miscellaneous costs (e.g., festival fees, prizes at recruitment events) were also documented.

### Analyses

Descriptive statistics, including counts, percent, means and standard deviations were used to summarize the data. Reach was assessed by the participation rate among screened and eligible residents. For representativeness, eligible and enrolled participants were descriptively compared to census data in the eight targeted counties [32]. Representative was also explored using t-tests (for comparing group means) and chi-square (for comparing proportions) to explore differences in three sub-group comparisons: 1) eligible participants who were identified using passive versus active strategies, 2) participants identified using passive strategies who enrolled versus those who did not enroll, and 3) participants identified using active strategies who enrolled versus those who did not enroll. Analyses were adjusted for the clustering of data by cohort. Cost data were described using sums and averages per participant screened and enrolled. To determine the range of cost per participant screened and cost per enrolled participant we compared information across the 8 counties (i.e., total dollars spent in the county divided by total number screened and then by total number enrolled).

## Results

**Fig. 1:**
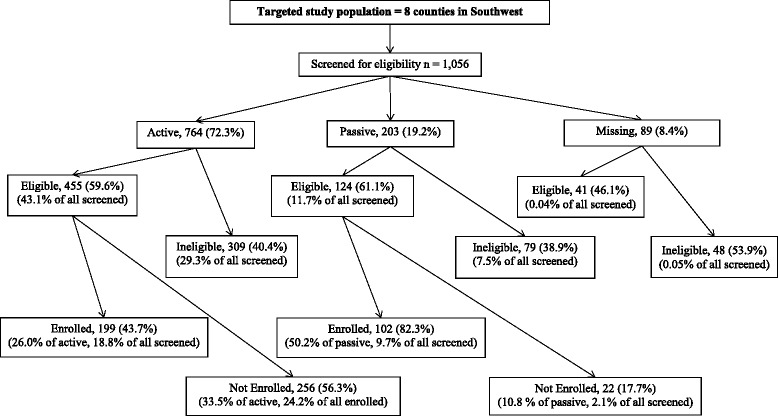
Flow of potential study participants through recruitment approaches from screening through study enrollment

In total, 1,056 participants were screened for eligibility, of which 620 (59%) were eligible, and 301 (49% of eligible) enrolled in the trial. Among the 301 recruited, 72% were identified using active methods, 19% were identified using passive methods, and 8% of screened individuals did not have recruitment strategy identified of which none enrolled in the trial (Fig. 1). A similar proportion of screened individuals met eligibility criteria (~60%) within both the active versus passive recruitment method categories. However, a higher proportion of eligible individuals screened when responding to passive methods enrolled in the trial (82%) when compared to those who were identified through active methods (44%). Nonetheless, when examining the overall sample, and when compared to passive recruitment methods, the active recruitment methods yielded considerably more screened (active = 764; passive = 203), more eligible (active = 455; passive = 124 individuals) and more enrolled (active = 199; passive = 102) individuals over the same period of time.

Seventy-seven percent of the enrolled participants were women, 90% were white, and 1% were Hispanic. The sample included 59% of participants with an education beyond high school, an average age of 40.1 (SD = 13.5) years and mean income of $21,981 (SD = 16,443; Table 2). The mean health literacy score for participants was 13.2 (SD = 2.4) on a 15-point scale. When compared to US Census data, the enrolled sample appeared to be representative in terms of age, race, ethnicity and years of schooling. However, men were underrepresented and the enrolled sample had a median income of less than half of US Census average for the region.

**Table 2:** Representativeness of enrolled participants to US census data and by active and passive recruitment strategies

Demo-graphic charac-teristics	U.S. census data (<65 years)	All screened *n* = 1,056^a^	Eligible *n* = 620^b^	Active recruitment			Passive recruitment			Cluster-adjusted t-test *p*-value		
				Eligible *n* = 455 (1)	Enrolled *n* = 199 (2)	Not Enrolled *n* = 256 (3)	Eligible *n* = 124 (4)	Enrolled *n* = 102 (5)	Not Enrolled *n* = 22 (6)	(1) vs. (4)	(2) vs. (3)	(5) vs. (6)
Female	48.1%	77.2%	77.3%	73.8%	77.9%	70.7%	87.9%	88.2%	86.4%	0.000	0.083	0.842
Mean Age (SD)	40.8	43.3 (14.6)	40.1 (13.5)	39.0 (13.2)	40.7 (12.9)	37.7 (13.3)	44.3 (14.0)	44.1 (14.0)	45.2 (14.5)	0.133	0.054	0.780
White	93.9%	88.8%	90.3%	89.5%	92.0%	87.5%	92.7%	95.1%	81.8%	0.178	0.302	0.169
Hispanic	1.6%	2.1%	1.4%	1.2%	0.5%	1.8%	1.6%	2%	0%	0.766	0.091	0.206
Beyond High school	57.9%	65.1%	60.4%	58.6%	64.3%	54.1%	72.6%	75.5%	59.1%	0.046	0.049	0.203
Mean Income (SD)	$48,105	$24,609 (17,651)	$21,981 (16,443)	$20,045 (15,257)	$19,548 (15,423)	$20,447 (15,141)	$29,073 (18,139)	$30,245 (18,177)	$23,636 (17,333)	0.002	0.635	0.077
Mean Health Literacy: Total (SD)^c^		13.3 (2.3)	13.2 (2.4)	13.2 (2.4)	13.3 (2.3)	13.1 (2.4)	13.5 (2.2)	13.7 (1.9)	13.0 (3.3)	0.190	0.389	0.433

^a^Missing recruitment method on 89 of 1056 participants screened (8.4%)

^b^Missing recruitment method on 41 of 620 participants who were eligible (6.6%)

^c^Based on a summed score out of three health literacy screening items (1 = low health literacy, 15-high health literacy)

When compared to active recruitment strategies, passive recruitment strategies yielded a higher proportion of eligible females (74% vs. 88%, *p* < 0.01), a higher proportion of eligible individuals with an education beyond high school (59% vs. 73%, *p* < 0.05), and eligible individuals with higher incomes ($20,044 vs $29,072, *p* < 0.01). However, there were no differences by age, race or ethnicity, or health literacy status when using active versus passive recruitment strategies. When comparing difference among those enrolled and not enrolled by recruitment method, there were few differences. One exception was that within the active recruitment group, enrolled participants were higher in the proportion with beyond high school education level compared to those who did not enroll (64% vs. 54%, *p* < 0.05). Within the actively recruited group, there were a few marginally significant differences (i.e., *p* < 0.10) in that enrolled participants were slightly more likely to be women and older while being less likely to be Hispanic when compared to those that declined participation. Within the passive recruited group, the average income of enrolled participants was marginally higher than the not enrolled participants ($30,245 vs $23,636, *p* = 0.08). When comparing the counties with the highest and lowest proportion of eligible participants enrolled, there were no clear patterns in terms of differences by recruitment strategies, IMU score, or rurality score.

The total recruitment costs for this trial was $60,566, of which $52,912 was active recruitment costs and $7,653 was passive recruitment costs (Table 3). When examining costs across the eight counties, the average cost was $58 per participant screened (range $32-88) and $213 per participant enrolled (range $55-415). When comparing the costs per enrolled participants across the counties, Wise ($415) and Washington ($321) had the highest recruitment costs per enrolled participant, whereas Lee ($55) and Montgomery ($114) had the lowest recruitment costs. The average cost ($278, range $90-$550) of an actively recruited and enrolled participant was over double the cost of a passively recruited and enrolled participant ($117 range $1-539).

**Table 3:** Recruitment costs by county, by active versus passive methods, and totals

	Lee	Giles	Pulaski	Washington	Grayson	Wise	Wythe	Montgomery	Total
Cost- Active strategies ($)									
community research assistant	1,762	0	1,800	379	1,442	272	1,165	303	7,123
university-affiliated research assistant	29	5,399	3,360	3,704	2,736	5,752	3,717	3,886	28,485
travel time	0	970	815	2,145	1,860	3,180	1,793	0	10,763
transportation	0	162	368	1,349	754	2,805	923	0	6,362
other (festival fees, prizes)	0	10.00	70	0	0	75	0	25	180
*Sub-total cost- Active strategies*	1,791	6,541	6,312	7,578	6,792	12,214	7,599	4,222	52,912
Cost- Passive strategies ($)									
community research assistant	75	0	37	8	58	0	31	8	217
university-affiliated research assistant	0	-^a^	68	72	107	130	90	0	467
Newspaper ads	389	597	320	0	525	924	977	0	3,732
Mailed postcards (to target pop)	0	0	128	2,613	235	131	131	0	3,238
*Sub-total cost- Passive strategies*	764	597	553	2,693	952	1,185	1,229	0	7654
Total cost- All strategies ($)	2,254	7,138	6,866	10,271	7,718	13,269	8,828	4,222	60,567
Cost per participant ($)									
Cost per screened participant	35	74	39	88	67	84	46	31	58
Cost per enrolled participant	55	230	130	321	221	415	221	114	213
Cost per participant screened via active strategies and enrolled	90	311	186	281	400	549	253	151	278
Cost per participant screened via passive strategies and enrolled	22	60	29	539	51	119	122	1	118

^a^missing recruitment method

## Discussion

This study addresses the current gap in the literature by providing a case report on the reach of a randomized controlled trial targeting reduced SSB intake in a rural 8 county region [25]. Populations, such as those that live in the Appalachia region of rural southwest Virginia, often represent the hardest groups to reach—and they remain underrepresented in research efforts [33, 34]. When considering the screened and recruited participants in the context of the census data presented in Table 2, it is clear that our study was over represented in women, but very similar across all other demographic comparators, with the exception of income level. Our recruitment strategies resulted in a sample with median income well below the census data for the region.

Similarly, as the larger trial has an explicit focus on health literacy strategies to reduce SSB intake, it was important to examine health literacy skills at screening. However, the current literature reveals a dearth of data on representativeness by health literacy status, as few behavioral trials assess health literacy among screened and enrolled participants [35]. Our findings suggest that there was no significant difference between eligible and enrolled participants and eligible but not enrolled participants for either the active or passive recruitment methods—suggesting representativeness in health literary. This could be due to the focus on health literacy and the use of plain language and clear communication techniques in the development of the recruitment materials [25]. Unfortunately, there are no national or state health literacy surveillance data to compare our study sample. However, we also used an objective measure of health literacy at enrollment (i.e., Newest Vital Sign [36]), and found that 33% of the enrolled participants had limited health literacy. This is consistent with a systematic review of 25 health literacy interventions [35], that estimated that about 38% of enrolled participants had low health literacy skills. Evaluating the health literacy status of both targeted and enrolled samples, as well as incorporating health literacy measures into current national surveillance efforts remains a key opportunity. Such advances would not only promote understanding of recruitment and program efforts, but would also help inform national health priority goals related to promoting health literacy and eliminating health disparities.

Our finding that participants recruited through active methods were more representative of the census data and screened and eligible groups is consistent with previous research that has tracked recruitment methods and compared characteristics to the population from which participants were recruited [9, 12, 17, 37–39]. While the active strategies yielded a lower income, education, and female sample, when comparing actively recruited individuals who were enrolled to actively recruited individuals that declined participation, those that enrolled were still more educated, less likely to be men, and more likely to have a higher average income (but not to the same level of those recruited through passive strategies). This suggests that additional strategies may be necessary to close the gap between screening of potentially eligible participants and enrollment rates.

Based on our study and systematic reviews of literature on recruitment strategies [12, 13, 40, 41] it is unlikely that there is a single recruitment strategy that can be generalized to all small communities. Indeed, across 8 counties, we found 8 unique combinations of recruitment strategies. Similarly, in the review of literature conducted by Uybico and colleagues, there was not a single recruitment strategy that was successful (i.e., had the highest proportional reach) in more than 50% of the studies [13]. Of note is that most studies that focus on recruitment have used quasi-experimental or descriptive study designs—including our study [13]. While the extant literature has some relatively consistent findings across studies (e.g., passively recruited participants are more likely to be enrolled), there is a need to have more definitive randomized trials that can clarify the most efficient methods to enroll difficult to reach participants. Based on our study and previous work, we recommend future studies to examine the processes by which recruitment methods can best be tailored to a given community and how this might be accomplished through community-engaged research approaches.

There are few studies on recruitment costs per enrolled participant across the literature, though this information is critical for research planning and for community organizations that will ultimately implement a program to reduce SSB. Even fewer studies examine cost of recruitment based on passive versus active recruitment strategies. However, in a study recruiting college students for a health promotion program found, similar to our investigation, that the overall cost of active recruitment was nearly double the cost of passive recruitment based on enrolled participants [18]. Further, regardless of the recruitment strategy used, ~$200 per enrolled participant appears to be efficient. For example, Raynor and colleagues documented a recruitment cost of ~ $500 per family recruited for a childhood weight management trial [19], Katula et al. reported ~ $400 per randomized community dwelling older adult [21]. Across weight gain prevention trials for young adults, costs were closer to those reported in our trial ranging from zero to ~ $1,100 per randomized participant, though the degree to which research staff time was captured across studies was unclear.

## Conclusion

Consistent with our hypotheses we found that participants recruited through passive versus active methods were more likely to be women, had higher average incomes, had higher educational attainment, and enrolled in the study at a higher rate. We also confirmed the hypothesis that recruitment strategies would vary by county. Indeed, tailoring of recruitment methods to community resources appears to be a necessary strategy as no two counties were identical. Finally, we replicated studies in other behavioral areas and documented that passive strategies were more cost-efficient but resulted in fewer total enrolled participants over the same period of time as active strategies. It also appears that passive strategies, based on the participation rate, reach a more motivated sample than active strategies.

Our results could have differential action recommendations for research and community organizations. For research projects, these data provide a method to document recruitment costs and to implement active recruitment strategies that are more likely to get a representative sample and, therefore, more generalizable results. Indeed, this is likely the most important finding for translational nutrition and physical activity scientists—passive strategies systematically seem to recruit participants that have higher socioeconomic status and are more educated—providing evidence that, when taken to scale, could exacerbate health disparities. In addition, our paper suggests that tailoring recruitment strategies for the locally available resources is feasible. It is unclear whether the same tailoring process could be used across behavioral and community contexts, though meeting with local stakeholders and determining how best to reach the targeted audience appears to be generalizable across communities and health behaviors. For community organizations implementing programs to reduce SSB, these data suggest that passive strategies are less costly and that a higher proportion of those screened will engage in the program [15, 18, 42]. This information is valuable when a community organization has limited resources to deliver intervention—identifying motivated participants that will engage in the resources is key for success. This, of course, needs to be balanced with the goal to reach a representative sample.

There are a number of limitations and future directions associated with our study. As noted earlier, this is not a randomized trial examining active and passive recruitment strategies [13]. Further, the combination of active and passive strategies within each county it difficult to determine if participants were exclusively influenced by one type of strategy over another [19]. Further, it is challenging to document a known denominator for passive recruitment strategies and, in some cases, active strategies. As a result, making a conclusion related to return on investment of one strategy over the other is problematic. Finally, our paper focuses on reach and not on the impact of active versus passive strategies on participant retention [18]. Despite these limitations we present data that is not typically available in the extant literature and provide new directions for research in the areas of tailoring recruitment strategies for individual communities and costs related to accruing participants.

## Abbreviations

IMU: Index of Medical Underservice
MUA: Medically Underserved Area
PD: Population Density (per square mile)
RS: Recruitment Strategy
SSB: Sugar-sweetened beverages
US: United States
WIC: Women, Infants, and Children
